# Prognostic significance of lymphocyte‐to‐monocyte ratio in diffuse large B‐cell lymphoma: a systematic review and meta‐analysis

**DOI:** 10.1002/2211-5463.12066

**Published:** 2016-05-04

**Authors:** Wen‐Kai Xia, Qing‐Feng Lin, Dong Shen, Zhi‐Li Liu, Jun Su, Wei‐Dong Mao

**Affiliations:** ^1^ Department of NephrologyThe Affiliated Jiangyin Hospital of Southeast University Medical CollegeJiangsuChina; ^2^ Department of OncologyThe Affiliated Jiangyin Hospital of Southeast University Medical CollegeJiangsuChina

**Keywords:** diffuse large B‐cell lymphoma, lymphocyte‐to‐monocyte ratio, meta‐analysis, prognosis

## Abstract

Published evidence on the prognostic significance of lymphocyte‐to‐monocyte ratio (LMR) in diffuse large B‐cell lymphoma (DLBCL) is controversial. We performed an updated meta‐analysis from 12 reports with 5021 patients to more accurately evaluate the prognostic value of LMR in DLBCL. Herein, we confirmed that patients with low LMR had shorter overall survival and progression‐free survival than those with high LMR in DLBCL. Subgroup analyses indicated that patient source, cut‐off values of LMR, treatment methods, and sample size showed similar prognostic performance in DLBCL patients. No significant heterogeneity was observed for progression‐free survival (PFS, *P* ^het^ = 0.192) among the enrolled studies. The meta‐analysis suggests that the LMR may be a potential biomarker in the prediction of clinical outcomes for DLBCL patients.

**Abbreviations**
ALC: absolute lymphocyte count
AMC: absolute monocyte count
CI: confidence interval
CRP: C‐reactive protein
DLBCL: diffuse large B‐cell lymphoma
HR: hazard ratio
LMR: lymphocyte‐to‐monocyte ratio
NLR: neutrophil‐to‐lymphocyte ratio
OS: overall survival
PFS: progression‐free survival
PLR: platelet‐to‐lymphocyte ratio

Diffuse large B‐cell lymphoma (DLBCL) is a major subtype of non‐Hodgkin lymphoma, responsible for over 25% of all newly incident patients across the world 1. Despite substantial improvement in treatment by the introduction of rituximab, long‐term survival of DLBCL remains poor due to its relapse and refractory after initial remission 2. A series of data have reported multiple biomarkers to predict clinical outcomes of DLBCL patients. However, the identification of high‐risk patients with expected 5‐year survival of less than 50% remains a great challenge with the use of traditional marker. Therefore, it is urgent to seek effective prognostic markers for the evaluation of a patient's prognosis using inexpensive, widely available, and easily explained clinical parameters.

Immunodeficiency is one of the strongest risk factors in adult non‐Hodgkin lymphoma 3. Systemic immune suppression is markedly associated with the occurrence of lymphoma 4. C‐reactive protein (CRP), neutrophil‐to‐lymphocyte ratio (NLR), and platelet‐to‐lymphocyte ratio (PLR) have been reported to predict long‐term survival in various solid cancers 5, 6, 7. In view of clinical application such as cost and technical limitations, recent studies have explored a surrogate biomarker, showing the host system immune status in peripheral blood and may serve as a prognostic indicator in DLBCL 8. The absolute lymphocyte count (ALC) and absolute monocyte count (AMC), surrogate markers of tumor microenvironment, have been reported as prognostic factors to predict outcomes of DLBCL patients 9.

Recent studies showed that the ALC/AMC (lymphocyte‐to‐monocyte ratio, LMR) is considered as a prognostic marker of tumor microenvironment in DLBCL patients 10, 11. For example, low LMR could reduce long‐term survival in patients with DLBCL 8, 10, 12, 13, 14. However, other investigators reported that the LMR is hardly correlated with survival in the germinal center‐type DLBCL patients treated with R‐CHOP 15. Moreover, a previous meta‐analysis, which enrolled nine studies, has suggested an increased risk with low LMR from a total of 4198 individuals 16. However, the following studies from 148 Taiwanese and 182 Serbian patients with newly diagnosed DLBCL exhibited no significant prognostic value in multivariate analysis 16, 17. These contradictory findings prompted us to explore more accurately the prognostic value of LMR in DLBCL patients. Herein, we performed an updated meta‐analysis including 12 studies to accurately estimate the effect of LMR on the survival of DLBCL patients using qualified relevant publications.

## Methods

### Publication selection

The published data were searched according to a literature review system with the Preferred Reporting Items for Systematic Reviews and Meta‐Analysis guidelines 18. Studies were identified in databases from PubMed and Web of Science (updated on March 3 2016) using the following terms: ‘lymphocyte’, ‘monocyte’, ‘ratio’, and ‘diffuse large B‐cell lymphoma’. Paper language was restricted to English. Only those published as full‐text articles were chosen as candidates.

### Inclusion and exclusion criteria

Studies evaluating the association between LMR and survival of DLBCL patients had to meet the following criteria: (a) explored the correlation of LMR with overall survival (OS) or progression‐free survival (PFS) of DLBCL patients; (b) sufficient information provided to estimate the hazard ratio (HR) and 95% confidence interval (CI) of OS or PFS; and (c) published in English.

### Data extraction

For each study, two reviewers (WKX and QFL) collected information carefully according to inclusion criteria, such as first author, publication year, study country, tumor stage, cut‐off value, treatment method, study design, follow‐up period, and sample size. HRs were extracted from multivariate analysis in the meta‐analysis. All disagreements about eligibility were resolved by discussion with another reviewer.

### Statistical analysis

stata software version 11.0 (College Station, TX, USA) was employed to analyze the extracted information. HRs with corresponding 95% CIs were used to assess the strength of association between LMR and the survival of DLBCL patients from multivariate analysis in each eligible study. Cochran's *Q* test and Higgins *I*‐squared statistic were performed to estimate the heterogeneity of pooled results. *I* ^2^ > 50% was regarded as significant heterogeneity. The random‐effects model (DerSimonian–Laird method) and fixed‐effects model (Mantel–Haenszel method) were employed to generate the pooled results. Stratified analyses were performed to investigate causes for the heterogeneity across studies. The stability of the combined results was evaluated by sensitivity analysis. Publication bias of studies was further evaluated by Egger's linear regression test. Statistical analyses were two‐sided and *P* < 0.05 was considered statistically significant.

## Results

### Characteristics of studies

**Figure 1:**
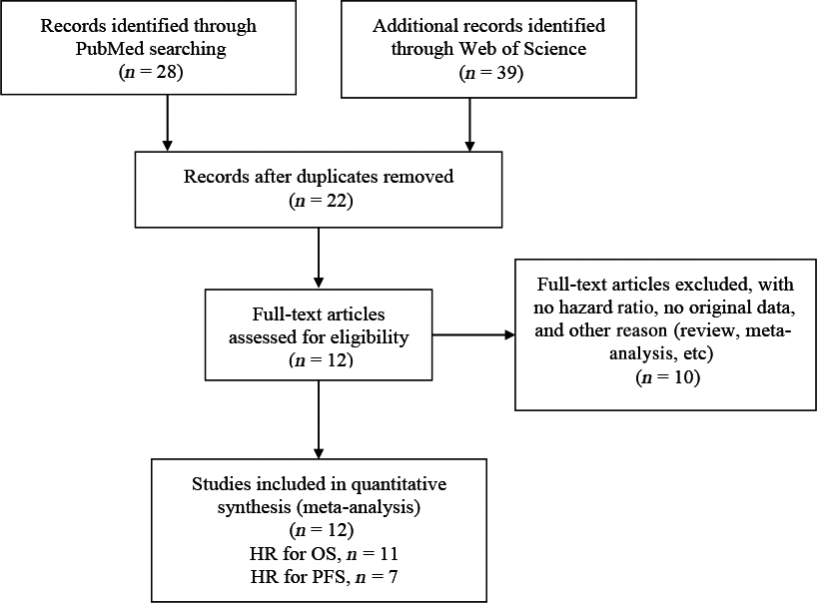
Flowchart of the eligible studies in this meta‐analysis.

As depicted in Fig. 1, according to the inclusion and exclusion criteria, 12 eligible studies were enrolled in this meta‐analysis. The characteristics of studies are shown in Table 1. Eligible studies with 5021 patients were enrolled. Seven studies consisted of two cohorts. One study was performed in Korea 8, Israel 19, Japan 13, Czech 20, and Taiwan 17, respectively. One study 14 included only late‐stage disease (III/IV). Eleven studies explored the association of LMR and OS, while seven studies investigated the correlation of LMR and PFS. The detail characteristics are summarized in Table 1.

**Table 1:** Characteristics of included studies

First author (publication year), country	Study design	LMR	Follow‐up (month/median)	Treatment received	Stage	No. of patients	Statistical method	Survival
Ho (2015), Taiwan 17	R	2.11	53.28	R‐CHOP	I–IV	148	Multivariate	OS, PFS
Jelicic (2015), Serbia 23	R	2.8	NR	R‐CHOP	I–IV	182	Multivariate	OS
Belotti (2015), Italy 26	R	2.4	24	R‐CHOP	I–IV	137	Multivariate	PFS
Prochazka (2014), Czech 20	R	2.43	36	R‐CHOP	I–IV	443	Univariate	OS
Koh (2014), Korea 8	R	3.04	37	R‐CHOP	I–IV	603	Multivariate	OS, PFS
Wei (2014), China 15	R	2.6	52	Non‐R‐CHOP	I–IV	168	Multivariate	OS, PFS
Tadmor (2014), Serbia 19	R	2.8	34	R‐CHOP	I–IV	222	Multivariate	OS
Markovic (2014), Israel and Italy 27	R	2.1	NR	R‐CHOP	I–IV	1017	Multivariate	OS
Li (2014), China 21	R	3.8	36	R‐CHOP	I–IV	244	Multivariate	OS, PFS
Watanabe (2013), Japan 13	R	4	58	R‐CHOP	I–IV	362	Multivariate	OS, PFS
Rambaldi (2013), Italy 14	R	2.6	77	Non‐R‐CHOP	III + IV	1057	Multivariate	OS
Li (2012), China 10	R	2.6	NR	R‐CHOP	I–IV	438	Multivariate	OS, PFS

R, retrospective; NR, not reported; Stage, Ann Arbor stage; PFS, progression‐free survival; OS, overall survival; Treatment methods describe whether the patients received R‐CHOP (rituximab, cyclophosphamide, doxorubicin, vincristine, and prednisone), or received non‐R‐CHOP, such as chemotherapy, radiotherapy, and surgery.

### Overall survival

**Figure 2:**
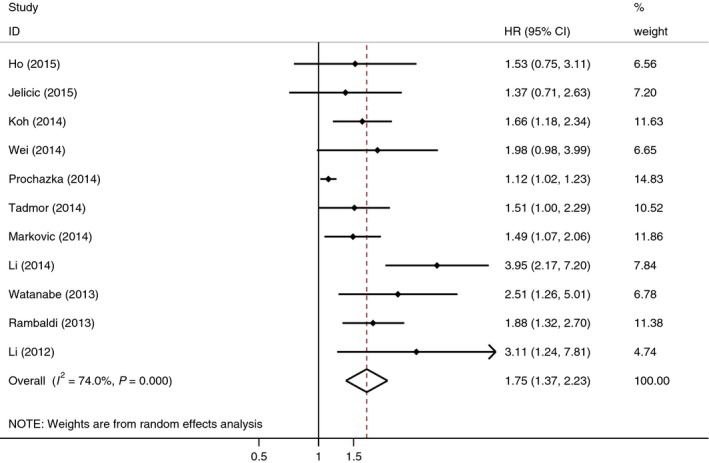
Forest plots of studies assessing HRs with corresponding 95% CIs of LMR for overall survival.

The overall results for OS are shown in Table 2. Eleven studies exhibited the association of LMR and OS in 4884 DLBCL patients. Results of the pooled analysis indicated that patients with low LMR were obviously associated with worse OS (HR = 1.75, 95% CI = 1.37–2.23, *P* < 0.001) with significant heterogeneity among these studies (*I* ^2^ = 74.0%; Fig. 2). In a stratified analysis by country, cut‐off value, treatment method, and sample size, a statistically significant association was observed for Western countries (HR = 1.41, 95% CI = 1.11–1.79) and Eastern countries (HR = 2.08, 95% CI = 1.65–2.63), LMR cut‐off < 3 (HR = 1.52, 95% CI = 1.21–1.91) and ≥ 3 (HR = 2.44, 95% CI = 1.41–4.22), R‐CHOP (HR = 1.72, 95% CI = 1.31–2.26) and non‐R‐CHOP (HR = 1.90, 95% CI = 1.38–2.61), and sample size < 400 (HR = 1.92, 95% CI = 1.51–2.44) vs. ≥ 400 (HR = 1.56, 95% CI = 1.16–2.08).

**Table 2:** The main results of the meta‐analysis

Variables	No. of studies	No. of patients	Regression model			
			Random	Fixed	*P* ^het^	*I* ^2^ (%)
OS	11	4884	1.75 (1.37–2.23)	1.27 (1.18–1.38)	< 0.001	74.0
**Stratified analysis**						
Country						
Western	5	2921	1.41 (1.11–1.79)	1.19 (1.10–1.30)	0.025	64.2
Eastern	6	1963	2.21 (1.61–3.02)	2.08 (1.65–2.63)	0.166	36.1
Cut‐off						
< 3	8	3675	1.52 (1.21–1.91)	1.22 (1.12–1.32)	0.014	60.2
≥ 3	3	1209	2.44 (1.41–4.22)	2.12 (1.61–2.79)	0.042	68.4
Treatment						
R‐CHOP	9	3659	1.72 (1.31–2.26)	1.24 (1.14–1.35)	< 0.001	75.0
Non‐R‐CHOP	2	1225	1.90 (1.38–2.61)	1.90 (1.38–2.61)	0.897	0.0
Sample size						
< 400	6	1326	1.97 (1.41–2.74)	1.92 (1.51–2.44)	0.115	43.5
≥ 400	5	3558	1.56 (1.16–2.08)	1.21 (1.12–1.32)	0.002	76.9
PFS	7	2100	2.31 (1.74–3.06)	2.21 (1.80–2.72)	0.192	31.0
**Stratified analysis**						
Country						
Western	1	137	8.00 (0.98–66.67)	8.00 (0.98–66.67)		
Eastern	6	1963	2.25 (1.71–2.97)	2.18 (1.77–2.69)	0.203	31.0
Cut‐off						
< 3	4	891	2.24 (1.31–3.82)	2.10 (1.37–3.21)	0.254	26.3
≥ 3	3	1209	2.41 (1.62–3.58)	2.24 (1.77–2.84)	0.205	27.9
Treatment						
R‐CHOP	6	1932	2.29 (1.68–3.13)	2.19 (1.77–2.70)	0.134	40.7
Non‐R‐CHOP	1	168	2.92 (0.99–8.61)	2.92 (0.99–8.61)		
Sample size						
< 400	5	1059	2.49 (1.55–3.99)	2.37 (1.74–3.23)	0.104	47.9
≥ 400	2	1041	2.08 (1.58–2.75)	2.08 (1.58–2.75)	0.429	0.0

OS, overall survival; PFS, progression‐free survival; *P* ^het^, *P* value for heterogeneity; Treatment methods describe whether the patients received R‐CHOP (rituximab, cyclophosphamide, doxorubicin, vincristine, and prednisone), or received non‐R‐CHOP, such as chemotherapy, radiotherapy, and surgery.

### Progression‐free survival

**Figure 3:**
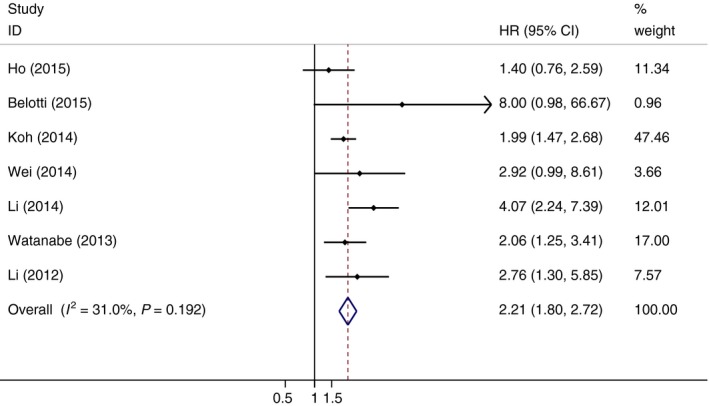
Forest plots of studies assessing HRs with corresponding 95% CIs of LMR for progression‐free survival.

The correlation of LMR and PFS in 2100 patients with DLBCL was further explored in the meta‐analysis (Table 2). The pooled data from seven studies showed that decreased LMR was significantly correlated with short PFS (HR = 2.21, 95% CI = 1.80–2.72, *P* < 0.001), and no heterogeneity was found among these studies (*I* ^2^ = 31.0%; Fig. 3). Subgroup analysis was further performed according to above confounders in OS. Stratification showed that low LMR was associated with poor prognosis in DLBCL patients regardless of study country, cut‐off value, therapeutic method, and sample size (Table 2).

### Test of heterogeneity

**Figure 4:**
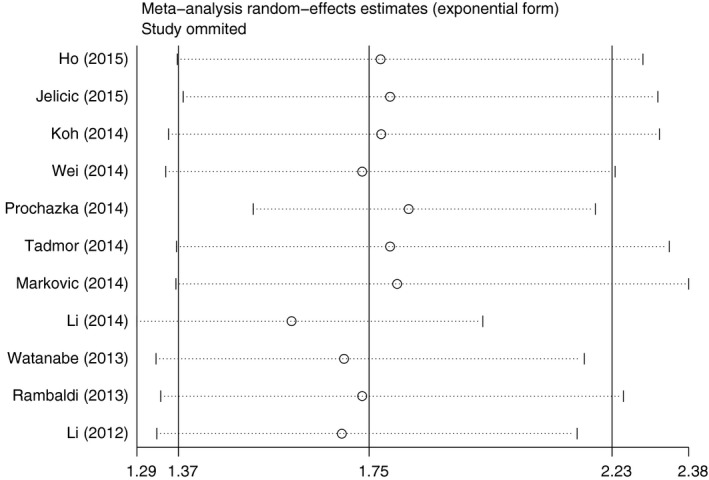
Sensitivity analysis of effect of individual studies on the pooled HRs for LMR and overall survival in DLBCL.

**Figure 5:**
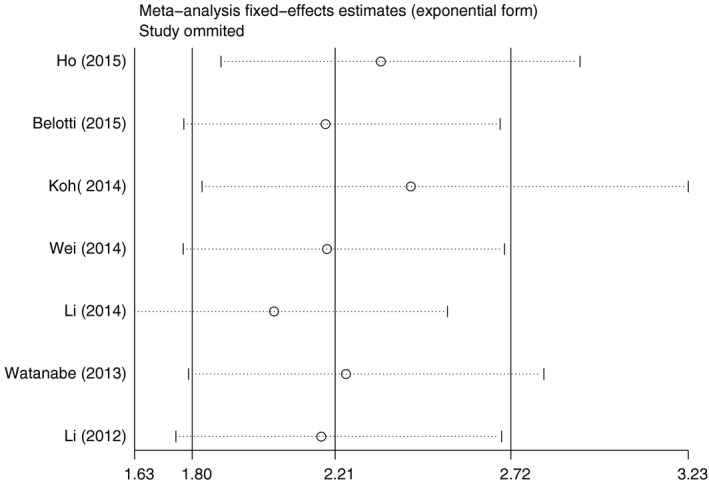
Sensitivity analysis of effect of individual studies on the pooled HRs for LMR and progression‐free survival in DLBCL.

There was no significant heterogeneity among studies for PFS (*P* ^het^ = 0.192) except for OS (*P* ^het^ < 0.001), and the random‐effect model was employed to estimate OS. Additionally, sensitivity analysis was conducted to further explore the source of heterogeneity and the stability of the results among studies for OS and PFS. A report by Li *et al*. 21 was the main origin of heterogeneity for OS, which the heterogeneity was markedly reduced after exclusion of these studies (*P* ^het^ = 0.069). The pooled results for OS and PFS was not significantly influenced by removing single study each time (Figs 4 and 5).

## Discussion

Mounting evidence shows a correlation between LMR and survival of DLBCL patients. However, these results remain controversial. In this updated meta‐analysis, associations between decreased LMR and survival of DLBCL patients were systematically evaluated. Our results demonstrated that DLBCL patients with low LMR had worse OS (HR = 1.75, 95% CI = 1.37–2.23) and PFS (HR = 2.21, 95% CI = 1.80–2.72) than those with high LMR. In stratified analysis by country, cut‐off value, treatment approach, and sample size, we observed that these confounders could not change prognostic performance of LMR in DLBCL patients. A previous meta‐analysis, which enrolled nine studies, showed an increased risk with low LMR from a total of 4198 individuals 16, which is consistent with our results. However, this previous meta‐analysis reported that study by Prochazka *et al*. 20 should be excluded due to its focus on elderly patients. Elderly patients are commonly not selected to enter clinical studies because of a higher incidence of deaths unrelated to lymphoma, but their complete remission rates are lower due to the suboptimal treatment 22. Our study provided a valuable adjunct to physician judgment by the inclusion of elderly patients with DLBCL. Meanwhile, the studies 17, 23 also exhibited the prognostic value of LMR in patients with newly diagnosed DLBCL. Therefore, this is an updated meta‐analysis of 12 published articles on the association between low LMR and clinical outcomes in DLBCL.

Recently, a series of investigations have reported the prognostic value of LMR in gastric cancer 7, lung cancer 24, and colorectal cancer 25. Furthermore, a few investigations reported the prognostic value of LMR in DLBCL patients. DLBCL patients with low LMR had markedly worse survival (OS and PFS) than those with high LMR 8. However, LMR was not correlated with long‐term survival in patients with germinal center‐type DLBCL 15. Lin *et al*. 16 suggested that the low LMR at diagnosis has an adverse effect on survival for patients with DLBCL based on the previous meta‐analysis from nine studies. In the present study, this was an updated meta‐analysis to explore the prognostic value of LMR in DLBCL patients and suggested that LMR was employed to evaluate clinical prognosis for patients with DLBCL. Additionally, LMR is a promising marker for clinical practice due to its inexpensive cost and routine test.

Several limitations should be acknowledged. First, the number of enrolled articles was relatively small. Studies of each subgroup were few by the stratified analyses. Second, LMR and clinical characteristics were not analyzed, such as bone marrow involvement, Ann Arbor stage, and lactate dehydrogenase. Finally, there is significant heterogeneity between OS and LMR; the results were relatively stable by sensitivity analysis, suggesting that the results were reliable.

In conclusion, decreased LMR is associated with poor prognosis in patients with DLBCL and shows an adverse effect on DLBCL patients, which could help clinicians stratify patients and select individual therapeutic strategy. Further studies are warranted to deeply understand the prognostic significance of LMR in DLBCL.

## Author contributions

WKX and WDM conceived and designed the experiments.WKX, QFL, DS, ZLL, and JS performed the experiments.WKX and QFL analyzed the data.DS and ZLL contributed reagents/materials/analysis tools. WKX and WDM wrote the manuscript.
